# Weekly Paclitaxel plus Capecitabine versus Docetaxel Every 3 Weeks plus Capecitabine in Metastatic Breast Cancer

**DOI:** 10.1155/2012/862921

**Published:** 2012-01-15

**Authors:** E. A. Wist, I. Mjaaland, E. Løkkevik, H. H. Sommer

**Affiliations:** ^1^Department of Oncology, Oslo University Hospital, P.O. Box 4956 Nydalen, 0424 Oslo, Norway; ^2^Department of Oncology and Hematology, Stavanger University Hospital, 4011 Stavanger, Norway

## Abstract

*Background*. We performed a randomized phase II study comparing efficacy and toxicity of weekly paclitaxel 80 mg/m^2^ (Weetax) with three weekly docetaxel 75 mg/m^2^ (Threetax), both in combination with oral capecitabine 1000 mg/m^2^ twice daily for 2 weeks followed by a 1-week break. *Patients*. Thirty-seven women with confirmed metastatic breast cancer were randomized. *Results*. Median TTF was 174 (Weetax) versus 147 days (Threetax) (*P=0.472*). Median OS was 933 (Weetax) versus 464 days (Threetax) (*P=0.191*). Reasons for TTF were PD 8/18 (Weetax), 9/19 (Threetax); and toxicity: 8/18 (Weetax), 8/19 (Threetax). ORR was 72% (Weetax) versus 26% (Threetax) (*P* = 0.01). The Threetax-combination resulted in a higher incidence of leuco-/neutropenia compared to Weetax. Grade II anemia was more pronounced in the Weetax group. No difference was found in quality of life. *Conclusion*. Taxanes in combination with capecitabine resulted in a high level of toxicity. Taxanes and capecitabine should be considered given sequentially and not in combination.

## 1. Introduction

Docetaxel 100 mg/m^2^ every 3 weeks has shown superiority in comparison with Methotrexate/5-FU/Leukovorin (MFL) [1] and mitomycin C/vinblastine (MV) [2] and has been recognized and recommended as second line chemotherapy in advanced metastatic breast cancer after failure on an anthracycline containing regimen. Paclitaxel is an effective agent in the treatment of metastatic breast cancer. In phase II and III trials where paclitaxel has been given every third week at doses of 135–250 mg/m^2^ administered by 3-hour or 24-hour infusion, response rates of 23–60% have been reported [3–7]. The activity and tolerability of weekly dosing of paclitaxel have attracted much interest. Weekly paclitaxel is usually associated with little myelosuppression and mild-to-moderate reversible neuropathy. It is considered as both dose-dense and dose-intense therapy and may, therefore, have advantages with respect to cumulative drug exposure. When given weekly by a 1-hour infusion in doses of 80–100 mg/m^2^, response rates of 21.5–68% have been reported [8–11]. In 2002 O'Shaughnessy et al. published the results of a phase III study comparing docetaxel alone with docetaxel in combination with capecitabine [12]. A superior TTP and overall survival achieved with the addition of capecitabine to docetaxel 75 mg/m^2^ indicated that this combination provides clear benefits over single-agent docetaxel 100 mg/m^2^. The side effects were described as manageable. Docetaxel/capecitabine therapy was described as an important treatment option for women with anthracycline-pretreated MBC [12]. In 2006 Blum et al. published the results of a phase II study combining capecitabine with weekly paclitaxel (80 mg/m^2^) as 1st line treatment for metastatic breast cancer [13]. The objective response rate was 55% and the regimen was described as active and tolerable as first-line therapy for women with MBC. We here describe a randomized phase II study comparing docetaxel every 3 weeks with weekly paclitaxel, both in combination with oral capecitabine. Time-to-treatment failure (TTF), response rates and quality of life were the main end points.

## 2. Material and Methods

Thirty-seven patients from 4 Norwegian hospitals were randomized to weekly paclitaxel 80 mg/m^2^ (Weetax) or docetaxel 75 mg/m^2^ every three weeks (Threetax) both in combination with oral capecitabine 1000 mg/m^2^ twice daily for 14 days followed by a 1-week break. Female patients age 18 years or older with histologically confirmed advanced or metastatic HER2 negative breast cancer with measurable or evaluable lesions were included. The chemotherapy was given as 1st or 2nd line chemotherapy. In the Weetax group 11/18 patients had one chemotherapy regimen for metastatic disease before inclusion compared to 12/19 patients in the Threetax group. Prior adjuvant chemotherapy was permitted. Concomitant bisphosphonate treatment was permitted. G-CSF was not given. Loperamid was used for treatment-related diarrhea.

Measurable disease was defined as at least one lesion that could be accurately measured in at least one dimension as ≥20 mm by conventional techniques, or as ≥10 mm by spiral CT scan. Lytic bone metastases as only site of recurrence were allowed. ECOG performance status 0–2 and a life expectancy of at least three months were required. The patients gave written informed consent and had to be accessible for treatment and follow-up. The patients were evaluated with physical examination, CT-scan of target lesions, chest X-ray, bone scan, X-ray of bones, and MRI. Other imaging techniques were used as appropriate. An objective evaluation of the treatment, limited to measurable/evaluable lesions, was performed every 9 weeks until progression. Follow-up after discontinuation of chemotherapy due to toxicity or at patient's request was carried out every 9 weeks until progression: thereafter every 6 months or at the discretion of the investigator. Objective response to treatment was evaluated according to RECIST criteria for soft tissue and visceral metastases [14] and according to WHO criteria for bone metastases [15]. Main end points were time-to-treatment failure (TTF) and Quality of life. Quality of life was measured at baseline and after 2, 4, 6, and 9 months, utilizing the EORTC QLQ-C30 (version 3) questionnaire [16].

 Secondary end points were response rates, overall survival (OS), safety, and quality of life.

The study was registered in http://clinicaltrials.gov/ct2/ show/NCT00201435?term=nct00201435&rank=1.

The Regional Ethics Committee, East Norway Health Region, authorized the study (REK 378-04-03133). Patients were informed that nonparticipation in the study would not in any way jeopardize their medical treatment.

## 3. Statistical Methods

To detect a difference of 20% in QOL between Weetax and Threetax 36 patients needed to be randomized. Time-to-treatment failure (TTF), response rate, overall survival, and toxicity were analysed. TTF and overall survival analysis included all patients and were calculated from the day of study entry until the day of documented treatment failure or death, respectively. Patients who died without documented progression were censored on the day of death or last follow-up. Patients who survived were censored on the day they were last known to be alive. TTF and overall survival were estimated using the Kaplan-Meier method.

## 4. Results

The patient characteristics are shown in Table 1. The groups were well balanced. Median time from diagnosis to recurrence was 33 months (CI 16–50) in the Weetax group and 55 months (CI 20–89) in the Threetax group (not significant).

**Figure 1:**
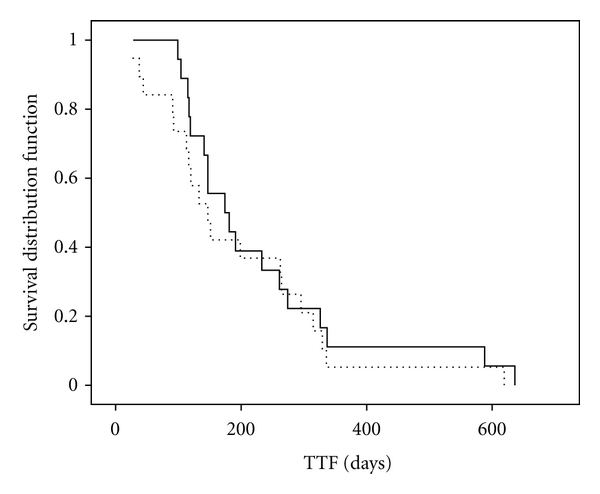
Kaplan/Meier analysis of time to treatment failure. Whole line: Weetax. Dotted line: Threetax. Median time to treatment failure: Weetax: 174 days. Threetax: 147 days (*P* = 0.472, n.s).

**Figure 2:**
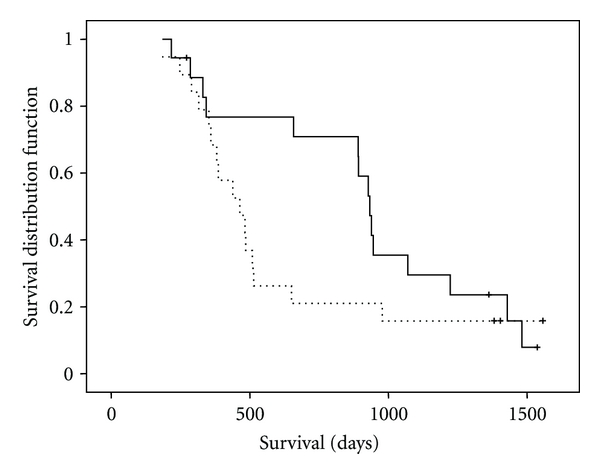
Kaplan/Meier analysis of overall survival. Whole line: Weetax. Dotted line: Threetax. Median overall survival: Weetax: 933 days. Threetax: 464 days (*P* = 0.191, n.s).

The treatment results are summarized in Table 2. Mean dose given of planned dose was docetaxel 94.6%, paclitaxel 100%, capecitabine 99.5% (Weetax), and 98.5% (Threetax). Overall response rate (CR + PR) was 13/18 (72%, W) versus 5/19 (26%, T) (*P* = 0.01). Two patients, one in each group, were not evaluable. Stable disease (SD) was seen in 3 patients in the paclitaxel group and in 9 patients in the docetaxel group. Progressive disease (PD) was seen in 1/18 (Weetax) versus 4/19 (Threetax). The difference is borderline significant (*P* = 0.052). Median TTF was 174 (Weetax) versus 147 days (Threetax) (*P* = 0.472, n.s.) (Figure 1). The main reasons for TTF were as follows; PD 8/18 (Weetax), 9/19 (Threetax); toxicity: 8/18 (Weetax), 8/19 (Threetax); and patient's request: 2/18 (Weetax), 2/19 (Threetax). Median OS was 933 (Weetax) versus 464 days (Threetax) (*P* = 0.191 n.s.) (Figure 2).

### 4.1. Toxicities

The Threetax-combination resulted in a significantly higher incidence of leucopenia and neutropenia compared to Weetax (Table 3). There was no significant difference with respect to Palmar-Plantar Erythrodysesthesia (PPE) and nail changes grade 3 and 4. In the Weetax group, however, there was more grade 2 toxicity with respect to PPE and nail changes.

Grade II anemia was significantly more pronounced in the Weetax-group.

### 4.2. Quality of Life

**Figure 3:**
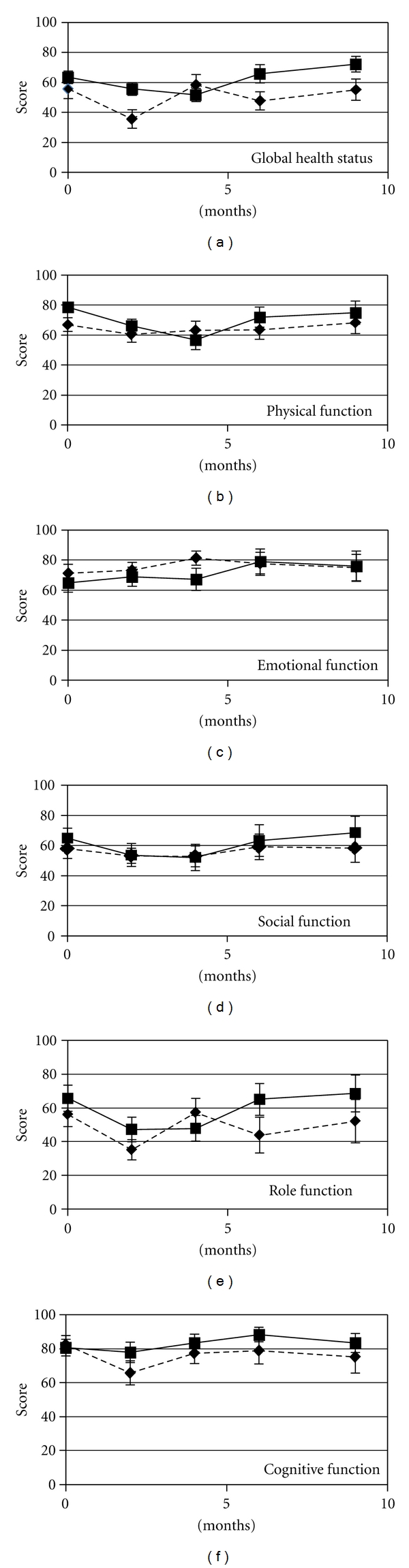
QLQ-C30 Global health status and functional scores.

**Figure 4:**
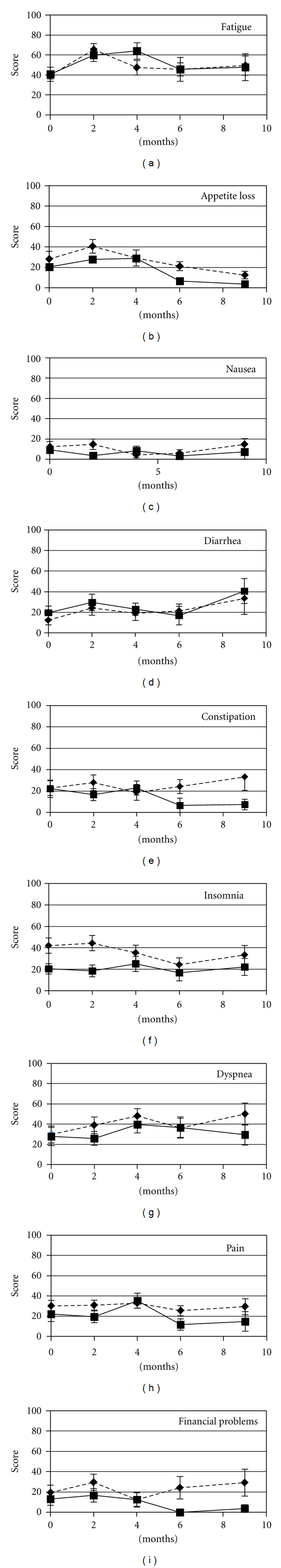
Figure 3. QLQ-C30 symptom scale scores and financial problems.

The results are presented in Figures 3 and 4. The physical, emotional, and cognitive functioning were at a high level in both groups. The role and social functioning were somewhat lower. There was no significant difference found between the groups. No statistical difference was found between the two regimens with respect to global score of QLQ-C30. Further, there were no differences with respect to appetite loss, nausea, constipation, diarrhea, insomnia, dyspnea, fatigue, and financial problems. The level of pain was lower in the Weetax-group compared to the Threetax group at baseline and at all measured time points except one. Insomnia had a higher—but not significant—score in the Threetax group.

## 5. Discussion

Time-to-treatment failure (TTF), rarely used as a primary end point, considers any reason for treatment interruption as an event in a Kaplan-Meier analysis (disease progression, treatment toxicity, patient preference, or death) whereas time to progression (TTP) focuses on progression of disease. Because TTF in its original definition is a composite end point that also includes subjective symptom assessment, it is seldom used for regulatory purposes. In spite of this TTF is the end point perhaps best reflecting the clinical situation. Our purpose was to perform a study that could be as close to the real life clinical situation as possible. We found that weekly paclitaxel and docetaxel every 3 weeks in combination with capecitabine resulted in stopping treatment due to toxicity and patients' preference in 10/18 in the paclitaxel group and 10/19 in the docetaxel group, that is, in more than 50% of the patients. This is a very high percentage especially taking into consideration that this was a first or second line regimen for treatment of metastatic breast cancer. The patients received more than 94% of the planned doses indicating that dose reduction was seldom performed due to hematological and nonhematological toxicity.

There was however significant more leuco-/neutropenia in the Threetax-group. This is as expected because weekly paclitaxel is associated with little bone marrow toxicity. In the Threetax-group more patients received palliative radiotherapy. This may have contributed to increased marrow toxicity.

The TTF for the two groups was 174 (Weetax) versus 147 days (Threetax), respectively. The TTF for Weetax is comparable to the TTP reported by O'Shaugnessy for the combination of docetaxel and capecitabine [12] (5, 8 versus 6, 1 months).

In spite of this lack of difference between the groups with respect to TTF, a significant difference in response rates was found. The response rate in the paclitaxel/capecitabine arm corresponds well with what was found by Blum et al. [13]. They reported a response rate of 55% and a clinical benefit of 65%. The response rate in the docetaxel/capecitabine arm was lower than that in the O'Shaugnessy study [12] (26% versus 42%), but the number of patients with stable disease was quite high in our study (9/19 patients). The median overall survival is twice as high in the Weetax group as compared with the Threetax group (933 versus 464 days), but the difference does not reach statistical significance. The weakness of this study is the small number of patients. The observed differences might therefore have been caused by chance. The main message would be that the combination of taxanes and capecitabine in the doses used in this study is too toxic for routine use. This is in line with Susnjar et al. who reported undue skin toxicity with a paclitaxel dose exceeding 60 mg/m^2^ when combined with capecitabine 1000 mg/m^2^ twice daily [17], whereas others (Uhlmann et al. [18] and Di Costanzo et al. [19]) recommended the doses used in our trial. The recent meta-analysis by Gennari and colleagues indicates that strategies for extending first-line chemotherapy are associated with a clinically modest but statistically significant improvement in OS and a clinically meaningful and statistically significant improvement in PFS [20]. They point out that we may be able to accomplish more by designing newer treatment sequences that use single agents in sequential fashion. This viewpoint is supported by Lidia Scapira commenting on this paper [21]. This is in accordance with our own opinion.

## 6. Conclusion

Based on the experiences from this study, we recommend that taxanes and capecitabine should be considered used in sequence instead of in combination.

## Figures and Tables

**Table 1:** Demographics and clinical characteristics of all randomised patients.

	Docetaxel + capecitabine	Paclitaxel + capecitabine
Median age (years)	52 (35–65)	53 (41–73)
Median weight (kg)	65	64.5
Median height (cm)	165	168.5
Median BSA (m^2^)	1.71	1.71
Performance status (ECOG)		
0	8	12
I	11	5
II	0	1
Estrogen receptor positive	12/19	12/18
Progesterone receptor positive	10/19	6/18
Prior postoperative radiotherapy	9/19	14/18
Prior radiotherapy for metastatic disease	7/19	2/18
Prior adjuvant chemotherapy	12/19	7/18
Prior chemotherapy for metastatic disease	12/19	11/18
Prior endocrine therapy for metastatic disease	14/19	12/18
Metastatic site		
Liver	7/19	9/18
Lung	5/19	7/18
Bone	6/19	10/18
>2 metastatic sites	4/19	1/18
Diabetes	2/19	0/18
Thromboembolism	0/19	1/18
Cardiovascular disease	0/19	1/18
Time from diagnosis to first recurrence (months) (CI)	55 (20–89)	33 (16–50)

**Table 2:** Treatment results.

	Docetaxel + capecitabine (T)	Paclitaxel + capecitabine (W)
Mean dose given (mg/m^2^) (% of planned dose)		
Docetaxel	75.6 (94.6)	
Paclitaxel		81.0 (101)
Capecitabine	1962.8 (98.1)	1990 (99.5)
Best response		
CR	0/19	1/18
PR	4/19	12/18
SD	10/19	3/18
PD	4/19	1/18
Not evaluable for response	1/19	1/18
Main Reason for TTF		
Disease progression	9/19	8/18
Toxicity	8/19	8/18
Patient's request	2/19	2/18
TTF days	147	174
OS days	464	933

**Table 3:** Incidence of drug-related toxicities grades 2 to 4.

Toxicity	Docetaxel + capecitabine		Paclitaxel + capecitabine		
	*N* = 19		*N* = 18		
	Grade I and II	Grade III and IV	Grade I and II	Grade III and IV	
Hematological					
Leukopenia	3 (15.8%)	9 (47.4%)	10 (55.6%)	0	*P* = 0.001
Neutropenia	0	12 (63.2%)	9 (50.0%)	3 (16.7%)	*p* = 0005
Febrile neutropenia	NA	1 (5.3%)	NA	1 (5,6%)	n.s.
Anemia	3 (15.8%)	0	14 (77.8%)	0	0.002
Non-hematological					
Infection	NA	1 (5.3%)	NA	2 (11.1%)	n.s.
Mucositis	9 (47.4%)	0	5 (27.8%)	0	N.s.
Pain	5 (26.3%)	0	6 (33.3%)	1 (5.6%)	n.s.
Fatigue	7 (36.8%)	1 (5.3%)	12 (67.7%)	1 (5.6%)	n:s
Diarrhoea	7 (36.8%)	0	3 (16.7%)	0	n.s.
Neuropathy, sensory	3 (15.8%)	0	7 (38.9%)	0	n.s.
PPE*	8 (42.1%)	1 (5.3%)	9 (50%)	4 (22.2%)	n.s.
Nail changes	7 (36.8%)	1 (5.3%)	11 (55.6%)	1 (5.6)	n.s.

*Palmar-Plantar Erythrodysesthesia.
